# Prevalence of hypertension among antiretroviral therapy naïve patients in Lagos, Nigeria

**DOI:** 10.1186/s40885-023-00253-6

**Published:** 2023-11-01

**Authors:** Oluwatosin Odubela, Nkiruka Odunukwe, Nasheeta Peer, Adesola Zaidat Musa, Babatunde Lawal Salako, Andre Pascal Kengne

**Affiliations:** 1 Department of Medicine, Faculty of Health Sciences, University of Cape Town, Cape Town, South Africa; 2 Clinical Sciences Department, Nigerian Institute of Medical Research, Yaba, Lagos, Nigeria; 3 Non-Communicable Diseases Research Unit, South Africa Medical Research Council, Cape Town, South Africa; 4 Monitoring and Evaluation Unit, Nigerian Institute of Medical Research, Yaba, Lagos, Nigeria; 5 Department of Medicine, University College Hospital, Ibadan, Oyo State, Nigeria

**Keywords:** Hypertension, Prevalence, ART-naïve, PLWH, HIV, Nigeria

## Abstract

**Background:**

The gains from successful antiretroviral therapy (ART) roll-out could be compromised by the increasing burden of non-communicable diseases, particularly cardiovascular diseases among people living with HIV (PLWH). Hypertension remains a significant contributor to cardiovascular diseases. This study aims to determine the prevalence and determinants of hypertension among ART-naïve PLWH in a large ART clinic in Lagos, Nigeria.

**Materials and methods:**

This study uses data collected from adult ART-naïve PLWH enrolled at an ART clinic over ten years. Participants aged 18 years and older, not pregnant, and not accessing care for post-exposure prophylaxis were included in the study. Hypertension was defined as systolic and diastolic blood pressure greater than or equal to 140 mmHg and 90 mmHg, respectively. Logistic regressions were used to investigate the factors associated with hypertension.

**Results:**

Among the 10 426 participants included in the study, the majority were females (66%) and aged 25—49 years (84%). The crude prevalence of hypertension was 16.8% (95%CI 16.4 – 17.2) while the age and sex standardised prevalence rate was 21.9% (95%CI 20.7 – 23.2), with males (25.8%, 95%CI 23.5 – 28.0) having a higher burden compared with females (18.3%, 95%CI 17.0 – 19.6). Increasing age, male gender, overweight or obesity, co-morbid diabetes mellitus or renal disease, and CD4 count ≥ 201 cells/μL were significantly associated with prevalent hypertension.

**Conclusion:**

There was a substantial burden of hypertension among ART-naïve PLWH, which was associated with the traditional risk factors of the condition. This highlights the need to integrate screening and care of hypertension into routine HIV management for optimal care of PLWH.

**Graphical Abstract:**

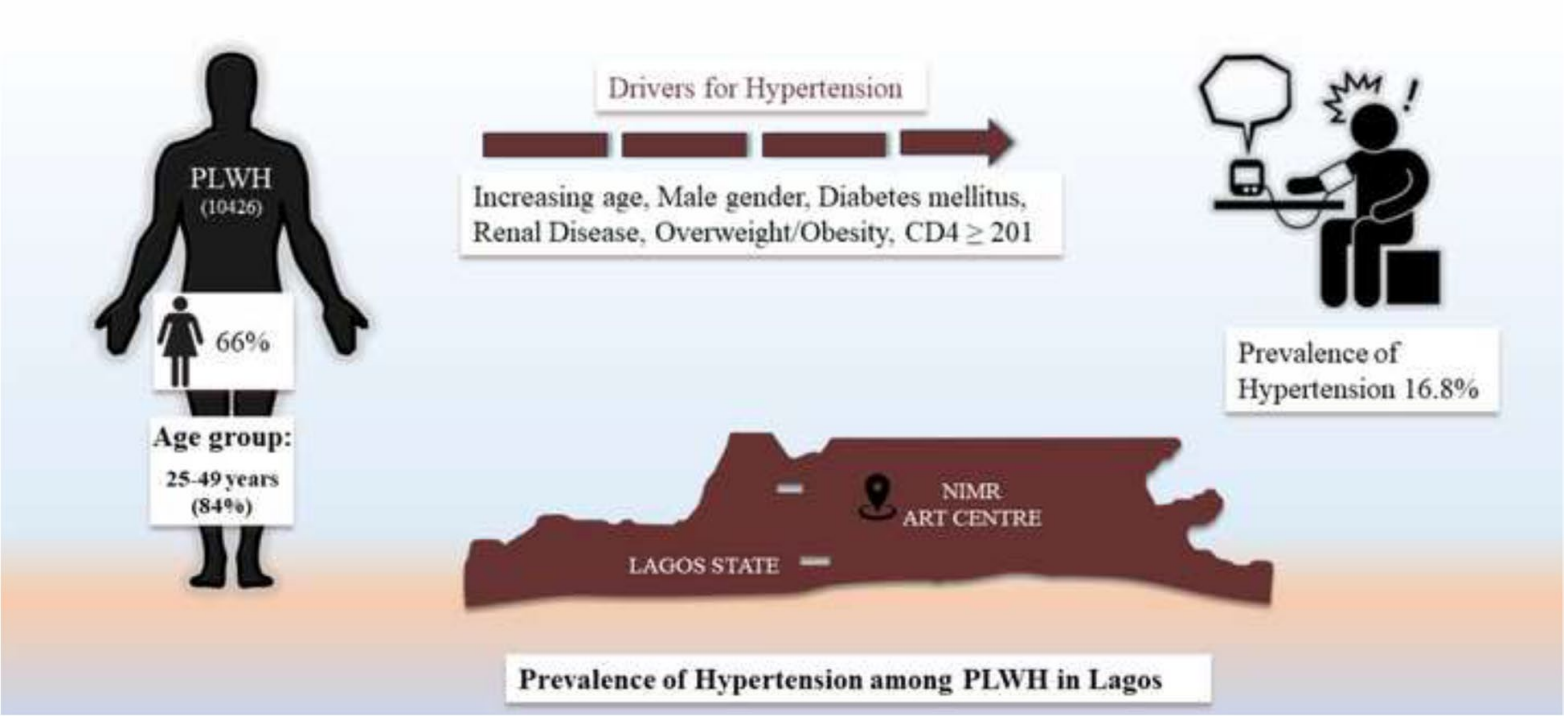

## Background

There have been improvements in Human Immunodeficiency Virus (HIV) care globally in recent times with decreased rates of HIV-associated mortality following the widespread availability and accessibility of antiretroviral therapy (ART) [1, 2]. The roll-out of ART to populations in low- and middle-income countries (LMICs), where more than two-thirds of people living with HIV (PLWH) reside, has led to increased longevity and survival in this population [3, 4]. However, this has been mitigated by the increasing burden of non-communicable diseases (NCDs) in this cohort [5–9] with rates similar to those not infected with HIV [10, 11]. Principal amongst these NCDs is cardiovascular disease (CVD) with hypertension a prominent contributor to its development. The burden of hypertension amongst PLWH worldwide was reported to be 24–25% [12] with a prevalence of 8.7 – 45.9% among PLWH in LMICs [12].

Despite the considerable burden of hypertension among PLWH, HIV care services are usually a stand-alone or parallel programme in most clinic settings in Sub-Saharan Africa (SSA) including Nigeria [13]. Consequently, PLWH incur additional costs [14, 15] such as out-of-pocket medical expenses, lost productive days due to illness, and transport costs when needing to separately access care for NCD co-morbidities [15]. These have increased the morbidity and mortality associated with NCDs among PLWH [16].

Nigeria, the most populated country in SSA, accounts for the largest population of PLWH in West Africa (1.9 million) and is second only to South Africa globally [17–19]. The prevalence of hypertension in this cohort, at 13 – 50% across studies in the country, is substantial [20–26]. However, most of the prevalence estimates were not age and sex-standardised, thus limiting generalization. This study describes the age- and sex- standardised prevalence and associated factors for hypertension among ART naïve PLWH at a large treatment centre in Lagos, Nigeria.

## Materials and methods

### Study setting

The Nigerian Institute of Medical Research (NIMR) is the foremost clinical research institute in Nigeria with a mandate to conduct research into diseases of public health importance. As part of her responsibilities, the Clinical Sciences Department within the institute conducts research in areas of communicable and non-communicable diseases in line with priorities and current health challenges. The Clinical Sciences Department operates an HIV clinic that has been in existence since 2002. The clinic was instituted as a research-based clinic to evaluate the effectiveness of generic ART in the country. This was commissioned under the auspices of the US President’s Emergency Plan for AIDS Relief (PEPFAR) program. The clinic has cumulatively enrolled more than 25 000 PLWH and caters to adults, pregnant women, and children infected with HIV. The clinic is in the economic capital of Nigeria, Lagos State providing care and services to a diverse population of citizens and foreign nationals based on the country’s National guidelines for HIV prevention, treatment, and care [27].

Care, treatment and management in the clinic is provided free of charge with support from the Nigerian government, PEPFAR and other multilateral agencies. Prior to enrollment, PLWH are offered counselling services (before and after conformation of HIV diagnosis) to provide reassurance and comfort as well as offer hope towards living positively with the HIV disease. In addition, information is also provided with respect to the ARVs (dosage, adherence, side effects and food -drug interactions).

Patient data relating to the clinical, therapeutic, and laboratory parameters are captured in an electronic database and maintained on a server onsite. Each patient is assigned a unique identification number, which is used to collate the clinic data. All patients enrolled in the clinic are offered a consent form to document their preference regarding the use of their biological samples and clinical data for research. Their responses do not affect their right to care and treatment in the clinic. The facility offers outpatient services only and patients requiring hospital admission are referred to facilities closest to their residence.

### Study design

This is a cross sectional study, reviewing data collected among ART-naïve PLWH enrolled in the ART clinic over 10 years (January 1, 2010 to December 31, 2019). Participants at enrollment into the clinic who were younger than 18 years, pregnant, or receiving care at a referral centre before transfer to the clinic were excluded. Paediatric participants upon attaining the age of 18 years, and those accessing care for post-exposure prophylaxis were also excluded from the study. All other participants enrolled in the clinic were included in the study analysis.

At enrollment, medical record officers collected sociodemographic information from prospective patients after counselling. The nurse thereafter evaluated specific clinical parameters (temperature, weight, height, blood pressure, and respiratory rate) of the patients. Weight was taken to the nearest 0.1 kg with the patient standing erect and barefoot on the weighing scale (Seca, GmBH). Height was measured in a similar posture with the marker placed on the crown of the head and readings taken to the nearest 0.1 cm (cm).

Blood pressure (BP) measurement was obtained with participants in a sitting position (for 5 min before the procedure) using a digital BP monitor, Omron M2 Eco model (HEM-7120-AF model, Omron Healthcare Co Ltd, Kyoto, Japan) with appropriate cuff size. The clinic adopted the use of the digital BP monitor as the standard equipment for BP readings in February 2014. Before the adoption of digital BP monitors, the manual sphygmomanometer was the standard equipment for BP measurement. Three BP readings were obtained at a sitting, and the average of the three readings was recorded as the participant's BP reading for the clinic consultation.

Clinical history was obtained, and physical examinations were conducted by the clinician. The WHO clinical staging for HIV infection [28] was assessed at the baseline clinic visit. Laboratory investigations include full blood count, cluster of differentiation 4 (CD4) count, viral load, serum urea and creatinine, random blood glucose, hepatitis B and C screening, and serum alanine aminotransferase.

Data collected for this study included the participants’ sociodemographic characteristics (age, sex, educational level, occupational and marital status), cardiovascular risk factors (body mass index [BMI], alcohol consumption), concurrent co-morbidities at enrollment (diabetes mellitus, hypertension, renal disease, tuberculosis), and HIV related factors (WHO clinical staging, viral load, CD4 counts). This was done to determine potential associations with (prevalent) hypertension at enrollment.

### Definitions

The study outcome of interest was prevalent hypertension among ART-naïve PLWH at enrollment. Hypertension was defined as systolic and/or diastolic blood pressure (SBP and DBP) greater than or equal to 140 mmHg and 90 mmHg, respectively [29]. In addition, prior history of hypertension diagnosis or use of anti-hypertensive medications, irrespective of current BP readings was defined as having hypertension [29]. The crude prevalence of hypertension was deduced by dividing the total number of PLWH with hypertension by the total study population, expressing the result in percentages. Similar methodology was applied to deduce annual prevalence of hypertension for the study, specific age groups and gender. The educational level attained by study participants was classified as none, primary, secondary, and tertiary. The first six years of formal education is termed primary, followed by another six years termed secondary (Junior Secondary School 1 – 3 and Senior Secondary School 1 -3), and finally tertiary (colleges of education, polytechnics, and university) levels of education [30]. Alcohol intake per week was categorized as nil, light ( 1–3 units), moderate (4—13 units), and heavy (≥ 14 units) [31]. Study participants were deemed to have co-morbid diabetes mellitus or renal disease if they provided a prior diagnosis (from clinical history or medical records) or were currently on treatment for the co-morbidity. Tuberculosis was deduced following both clinical history and physical examination [32, 33]. BMI was calculated as the weight (in kilogram) divided by height squared (in metres squared) – Kg/m^2^. BMI was classified as underweight (< 18.5), normal (18.5 – 24.9), overweight (25.0 -29.9), and obese (≥ 30.0) [34]. Viral suppression was defined as a viral load below 1000 copies per millimeter [35] and CD4 counts were classified per CDC Staging [36, 37].

### Statistical analysis

Data are presented as counts and percentages for categorical variables, while mean and standard deviations or median and 25th—75th percentiles were used for continuous variables. The crude prevalence of hypertension was determined from the blood pressure readings in the study while age and sex standardised prevalence was computed using the Nigeria HIV/AIDS Indicator and Impact Survey (NAIIS) 2018 report [19]. The NAIIS 2018 report is the latest version of the household survey of HIV infection in the country and direct standardization method was employed to determine the age and sex standardised prevalence in our study. Logistic regression analysis (odds ratio and 95% Confidence Intervals) was used to determine the associations with hypertension. Variables found to be statistically significant in univariate analysis (p-value < 0.05) were included in the regression analysis for the multivariable regression models (enter and backward elimination methods). The backward elimination method was used select significant variables in the final multivariable model based with the Akaike information criterion (AIC) determined for the selected model. Statistical significance was set at a *p*-value of < 0.05. Data were analysed using Statistical Package for Social Sciences (SPSS) version 26 (IBM SPSS Inc, Chicago, IL).

## Results

Of the 11 281 PLWH who had baseline clinic visits recorded in the database during the 10-year study period, 10 426 PLWH (92.4%) fulfilled the inclusion criteria and were included in the analysis. Table 1 depicts the sociodemographic characteristics of study participants by gender. The mean age at enrollment was 36.4 (± 9.3) years with a higher mean age among males compared to females [40.4 (± 9.4) vs 34.4 (± 8.6) years, *p* < 0.001]. Most participants were aged 25 – 49 years (84%) and there was a preponderance of females (66%) in the study.

**Table 1:** Sociodemographic characteristics and clinical presentation of ART-naïve PLWH by gender at enrollment

Characteristics	Overall (*n* = 10,426)	Female (*n* = 6886)	Male (*n* = 3540)	*p*-value
^a^Age in years (± SD)	36.41 (± 9.34)	34.38 (± 8.61)	40.37 (± 9.43)	< 0.001
Age groups in years, n (%)				< 0.001
18 – 24	652 (6.3)	532 (7.7)	120 (3.4)	
25—49	8756 (84.0)	5899 (85.7)	2857 (80.7)	
≥ 50	1018 (9.7)	455 (6.6)	563 (15.9)	
Educational Status, n (%)				< 0.001
None	507 (4.9)	397 (5.8)	110 (3.1)	
Primary	1941 18.8)	1261 (18.5)	680 (19.4)	
Secondary	4485 (43.5)	2960 (43.5)	1525 (43.5)	
Tertiary	3380 (32.8)	2191 (32.2)	1189 (34.0)	
Occupation, n (%)				< 0.001
Unemployed	1206 (11.6)	941 (13.7)	265 (7.5)	
Employed	9093 (87.6)	5890 (85.8)	3203 (90.9)	
Retired	87 (0.8)	30 (0.5)	57 (1.6)	
Marital status, n (%)				< 0.001
Single	2986 (28.6)	1976 (28.7)	1010 (28.5)	
Married	5879 (56.4)	3676 (53.4)	2203 (62.3)	
Separated	633 (6.1)	764 (11.1)	164 (4.6)	
Widowed	928 (8.9)	470 (6.8)	163 (4.6)	
Alcohol intake, n (%)				< 0.001
Never	7339 (77.3)	5416 (86.4)	1923 (59.7)	
Light and Moderate	1978 (20.8)	833 (13.3)	1145 (35.6)	
Heavy	172 (1.6)	20 (0.3)	152 (4.7)	
Co-morbidity, n (%)				
Tuberculosis	581 (5.6)	306 (4.4)	275 (7.8)	< 0.001
Diabetes Mellitus	80 (0.8)	33 (0.05)	47 (1.3)	< 0.001
Renal disease	35 (0.3)	16 (0.2)	19 (0.5)	0.001
Hypertension	358 (3.4)	196 (2.8)	162 (4.6)	< 0.001
Mean Systolic BP (± SD)	114.20 (± 19.91)	112 02 (± 19.22)	118.45 (± 20.53)	< 0.001
Mean Diastolic BP (± SD)	73.20 (± 12.93)	72.34 (± 12.67)	74.88 (± 13.26)	< 0.001
BMI categories (kg/m^2^), n (%)				
Median (25th – 75th percentiles)	23.12 (20.28 – 26.63)	23.44 (20.32 – 27.12)	22.58 (20.18 – 25.54)	
Underweight/Normal	5735 (64.8)	3606 (61.7)	2129 (70.6)	0.009
Overweight/Obese	3122 (35.2)	2237 (38.3)	885 (29.4)	
WHO Stage, n (%)				< 0.001
1	1347 (19.8)	1007 (22.5)	340 (14.6)	
2	2471 (36.4)	1683 (37.7)	788 (33.8)	
3	2287 (33.6)	1387 (31.0)	900 (38.6)	
4	693 (10.2)	393 (8.8)	300 (12.8)	
Viral Load (copies/ml), n (%)				< 0.001
≤ 1000	2458 (28.9)	1677 (29.5)	781 (27.9)	
1001 – 100,000	3004 (35.4)	2197 (38.6)	807 (28.8)	
≥ 100,001	3029 (35.7)	1814 (31.9)	1215 (43.3)	
CD4 count (cells/μL), n (%)				< 0.001
≤ 200	4483 (44.8)	2739 (41.4)	1744 (51.4)	
201 – 499	3985 (39.8)	2711 (41.0)	1274 (37.6)	
≥ 500	1537 (15.4)	1164 (17.6)	373 (11.0)	

*BP* Blood pressure, *SD* Standard deviation, *BMI* Body Mass Index, *Underweight/Normal* ≤ 24.9 kg/m^2^, *Overweight/Obese* ≥ 25.0 kg/m^2^, *CD4* Cluster of differentiation 4

^a^Mean age and Standard deviation. Educational Status; Primary (First six years of formal education), Secondary (Second six years of formal education), and Tertiary (college of education, polytechnic, university). Alcohol intake; Never refers to participants who have never taken alcohol, while light, moderate and heavy refer to participants who consume 1—3 units, 4—13 units, and ≥ 14 units of alcohol respectively per week. Co-morbid diagnosis of hypertension, diabetes mellitus, and renal disease was made from history taking and self-report by study participants. Co-morbid diagnosis of tuberculosis was made through a combination of history taking and clinical examination

At enrollment, a majority of PLWH were employed (87.6%), had at least secondary school level of education (76.3%), were married (56.4%), had never drunk alcohol (77.3%), and had normal or underweight (64.8%) body mass index (BMI). Most participants had CD4 counts less than 500 cells/μL (84.6%), were not virally suppressed (71.1%), and were classified as either WHO clinical stages 1 or 2 (56.2%) irrespective of gender. Tuberculosis (5.6%) was the leading co-morbidity with similar distribution between men and women. Mean BP was 114/73 mmHg with higher levels in men (118/75 mmHg) compared to women (112/72 mmHg) for both SBP and DBP (*p* < 0.001).

**Fig. 1:**
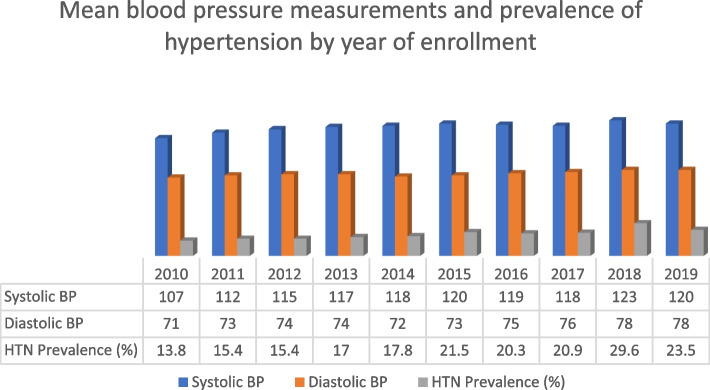
Mean Blood pressure measurements and prevalence of hypertension among ARV-naïve PLWH by year of enrollment

The mean systolic and diastolic blood pressure (SBP and DBP) for each year of enrollment is displayed in Fig. 1. The lowest mean SBP reading was recorded in 2010 (107 mmHg) while the highest mean SBP reading was in 2018 (123 mmHg). The highest mean DBP was recorded in 2018 and 2019 (78 mmHg) with the lowest value for mean SBP in 2010 (71 mmHg). The highest prevalence of hypertension was noted in 2018 (29.6%) while the lowest was recorded in 2010 (13.8%).

**Fig. 2:**
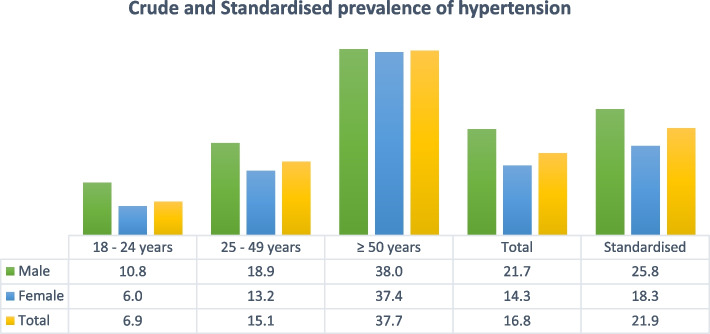
Crude and standardised prevalence of hypertension among ART-naïve PLWH

The crude as well as age and sex standardised prevalence of hypertension is shown in Fig. 2. The crude prevalence of hypertension was 16.8% (95%CI 16.4 – 17.2) overall with higher rates in men (21.7%, 95%CI 20.3 – 23.1) compared with women (14.3%, 95%CI 13.5 – 15.2); *p* < 0.001. The age and sex standardised prevalence rate for hypertension was 21.9% (95%CI 20.7—23.2) overall, 25.8% (95% CI 23.5—28.0) in men, and 18.3% (95%CI 17.0 – 19.6) in women. The highest prevalence of hypertension was among those ≥ 50 years of age (37.7%).

The characteristics of participants by hypertension status are presented in Table 2. PLWH with hypertension, when compared to those without hypertension, were found to be older (41.5 vs. 35.4 years, *p* < 0.001) and more likely to be overweight or obese (52.3% vs. 31.8%, *p* < 0.001). Among participants with hypertension, 75.5% (*n* = 1321) were aged between 25 and 49 years. All other sociodemographic and clinical characteristics among study participants were similar irrespective of hypertension status.

**Table 2:** Sociodemographic and clinical characteristics of ARV-naïve PLWH with or without hypertension with univariable and multivariable logistic regression

Characteristics	Hypertension present (N, 1750), n (%)	Hypertension absent (N, 8676), n (%)	Odds ratio (95% CI)	*p*-value	Adjusted Odds ratio (95% CI) (Enter method)	*p*-value	Adjusted Odds ratio (95% CI) (Backward elimination)	*p*- value
^a^Mean Age in years (± SD)	41.54 (± 10.08)	35.38 (± 8.83)	1.07 (1.06 – 1.07)	< 0.001				
Age groups in years, n (%)				< 0.001		< 0.001		< 0.001
18—24	45 (2.6)	607 (7.0)	1.00		1.00		1.00	
25—49	1321 (75.5)	7435 (85.7)	2.40 (1.76 – 3.26)		1.51 (0.91 – 2.49)		1.49 (0.91 – 2.43)	
≥ 50	384 (21.9)	634 (7.3)	8.17 (5.88 – 11.34)		3.85 (2.22 – 6.68)		3.81 (2.22 – 6.53)	
Gender, n (%)				< 0.001		< 0.001		< 0.001
Female	983 (56.2)	5903 (68.0)	1.00		1.00		1.00	
Male	767 (43.8)	2772 (32.0)	1.66 (1.50 – 1.84)		1.71 (1.43 – 2.05)		1.77 (1.49 – 2.09)	
Marital Status, n (%)				< 0.001		0.001		0.001
Single	347 (19.8)	2639 (30.4)	1.00		1.00		1.00	
Married	1056 (60.3)	4823 (55.6)	1.67 (1.46 – 1.90)		1.29 (1.05 – 1.59)		1.28 (1.04 – 1.57)	
Separated	126 (7.2)	507 (5.8)	1.89 (1.51 – 2.37)		1.35 (0.93 – 1.97)		1.35 (0.93 – 2.60)	
Widowed	221 (12.6)	707 (8.1)	2.38 (1.97 – 2.87)		1.92 (1.40 – 2.65)		1.90 (1.39 – 2.60)	
Educational Status, n (%)				0.003		0.524		
None	87 (5.0)	420 (4.9)	1.00		1.00			
Primary	350 (20.2)	1591 (18.5)	1.06 (0.82 – 1.38)		1.19 (0.77 – 1.84)			
Secondary	683 (39.5)	3802 (44.3)	0.87 (0.68 – 1.11)		1.02 (0.67 – 1.55)			
Tertiary	611 (35.3)	2769 (32.3)	1.06 (0.83 – 1.36)		1.03 (0.67—1.57)			
Occupation, n (%)				< 0.001		0.910		
Unemployed	144 (8.2)	1062 (12.3)	1.00		1.00			
Employed	1569 (89.8)	7524 (87.1)	1.54 (1.28 – 1.85)		0.94 (0.71 – 1.26)			
Retired	35 (2.0)	52 (0.6)	4.96 (3.13 – 7.88)		1.01 (0.48 – 2.13)			
Alcohol intake, n (%)				< 0.001		0.396		
Never	1140 (72.6)	6199 (78.3)	1.00		1.00			
Light and Moderate	393 (25.0)	1585 (20.0)	1.35 (1.19 – 1.53)		1.13 (0.93 – 1.37)			
Heavy	38 (2.4)	134 (1.7)	1.54 (1.07 – 2.22)		1.24 (0.72 – 2.11)			
Co-morbidity, n (%)								
Diabetes Mellitus	39 (2.3)	41 (0.5)	4.81 (3.09 – 7.48)	< 0.001	2.63 (1.38 – 5.03)	0.003	2.59 (1.37 – 4.93)	0.004
Renal disease	19 (1.1)	16 (0.2)	5.96 (3.06 – 11.62)	< 0.001	4.20 (1.04 – 16.93)	0.044	4.20 (1.04 – 16.89)	0.043
Tuberculosis	68 (3.9)	513 (5.9)	0.64 (0.50 – 0.83)	0.001	0.60 (0.39 – 0.93)	0.023	0.59 (0.38 – 0.90)	0.015
BMI categories (kg/m^2^), n (%)								
^a^Median (25th – 75th percentile)	25.26 (22.43 – 28.97)	22.72 (19.98 – 26.12)		< 0.001				
Underweight/Normal	700 (47.7)	5035 (68.2)	1.00	< 0.001	1.00	< 0.001	1.00	< 0.001
Overweight/Obese	769 (52.3)	2353 (31.8)	2.37 (2.12 – 2.65)		2.15 (1.81 – 2.54)		2.18 (1.85 – 2.57)	
WHO Stage, n (%)				< 0.001		0.629		
1 – 2	715 (61.0)	3103 (55.2)	1.00		1.00			
3 – 4	458 (39.0)	2522 (44.8)	0.79 (0.69 – 0.90)		0.96 (0.80—1.14)			
Viral Load (copies/ml), n (%)				< 0.001		0.25		
≥ 1001	938 (65.5)	5095 (72.2)	1.00		1.00			
≤ 1000	495 (34.5)	1963 (27.8)	1.37 (1.21 – 1.55)		1.11 (0.93 – 1.31)			
CD4 count (cells/μL), n (%)				< 0.001		0.025		0.012
≤ 200	618 (36.3)	3865 (46.5)	1.00		1.00		1.00	
≥ 201	1083 (63.7)	4439 (53.5)	1.53 (1.37 – 1.70)		1.21 (1.03 – 1.44)		1.23 (1.05 – 1.45)	

*SD* Standard deviation, *BMI* Body Mass Index, *Underweight/Normal* ≤ 24.9 kg/m^2^, *Overweight/Obese* ≥ 25.0 kg/m^2^, *CD4* Cluster of differentiation 4

^a^Mean age and Standard deviation. Educational Status; Primary (First six years of formal education), Secondary (Second six years of formal education), and Tertiary (college of education, polytechnic, university). Alcohol intake; Never refers to participants who have never taken alcohol, while light, moderate and heavy refer to participants who consume 1—3 units, 4—13 units, and ≥ 14 units of alcohol respectively per week. Co-morbid diagnosis of diabetes mellitus and renal disease was made from history taking and self-report by study participants. Co-morbid diagnosis of tuberculosis was made through a combination of history taking and clinical examination

The univariable and multivariable analysis are also displayed in Table 2. Compared with those aged 18 – 24 years, participants aged ≥ 50 years (OR: 8.17, 95% CI: 5.88 – 11.34) and 25—49 years (OR: 2.40, 95% CI: 1.76 – 3.26) were more likely to have hypertension. Furthermore, being male (OR: 1.66, 95% CI: 1.50 -1.84), widowed (OR: 2.38, 95%CI: 1.97 – 2.87), retired (OR: 4.96, 95%CI: 3.13 -7.88), overweight or obese (OR: 2.37, 95%CI: 2.12 – 2.65), and having co-morbid self-reported diabetes mellitus (OR: 4.81, 95%CI: 3.09 – 7.48), or renal disease (OR: 5.96, 95%CI: 3.06 – 11.62) were associated with higher odds of hypertension. PLWH with favourable HIV laboratory parameters at enrollment (WHO clinical stage 1 and 2, Viral load ≤ 1000 copies/ml, and CD4 counts ≥ 201cells/µL) were also found to have higher odds of hypertension. However, having co-morbid tuberculosis was significantly associated with decreased odds of hypertension.

The multivariable regression model (Enter method) included age groups, sex, marital status, education, occupation, BMI categories, alcohol intake, co-morbidities (diabetes mellitus, renal disease, and tuberculosis), and HIV related factors (WHO staging of HIV, viral load, and CD4 counts). Male gender (AOR: 1.71, 95%CI: 1.43 – 2.05), age group ≥ 50 years (AOR: 3.85,95%CI: 2.22 -6.68), and marital status [married (AOR: 1.29, 95%CI: 1.05 – 1.59) and widowed (AOR: 1.922, 95%CI: 1.40 – 2.65)] were significant sociodemographic characteristics associated with hypertension. Participants who were overweight or obese (AOR: 2.15, 95%CI: 1.18 – 2.54), had diabetes mellitus (AOR: 2.63, 95%CI: 1.38 – 5.03), renal disease (AOR: 4.21, 95%CI: 1.04 – 16.98) and CD4 counts ≥ 201cells/µL (AOR: 1.21, 95%CI: 1.03 – 1.44) were more likely to have hypertension. Participants with tuberculosis (AOR: 0.60, 95%CI: 0.39 – 0.93) had lower odds of hypertension.

The backward elimination method was employed to select the best fit model to identify determinants associated with hypertension in the study. Age group, sex, marital status, BMI categories, co-morbidities (diabetes mellitus, renal disease, and tuberculosis), and CD4 counts were variables included in the model using backward elimination method. Male gender (AOR: 1.77, 95%CI: 1.49 – 2.09), age groups 25 – 49 years and ≥ 50 years (AOR: 1.49, 95%CI: 0.91 – 2.43 and AOR: 3.81, 95%CI: 2.22 – 6.53 respectively), and marital status were significant sociodemographic characteristics associated with hypertension. Participants who were overweight or obese (AOR: 2.18, 95%CI: 1.85 – 2.57), had diabetes mellitus (AOR: 2.59, 95%CI: 1.37 – 4.93), renal disease (AOR: 4.20, 95%CI: 1.04 – 16.89) and CD4 counts ≥ 201cells/μL (AOR: 1.23, 95%CI: 1.05 – 2.57) were more likely to have hypertension. Participants with tuberculosis (AOR: 0.59, 95%CI 0.38 – 0.90) had lower odds of hypertension (Table 2). The AIC for the selected model was 574.437 (*p* < 0.001).

## Discussion

With over one in five PLWH having hypertension, this study highlights the substantial burden of this condition among ART-naïve PLWH at one of the largest HIV clinics in Nigeria. Majority of participants with hypertension were aged 25—49 years, i.e., in the economically active age group. Notably, traditional risk factors of older age, overweight or obesity, diabetes mellitus, and renal disease were associated with prevalent hypertension. HIV factors of low CD4 count and high viral load, which lead to poorer outcomes, were not related to the burden of hypertension.

The age-standardised hypertension prevalence of 21.9% among ART-naïve PLWH in this study seems to track the 25.8% reported in the general population in Nigeria [38]. Thus, this suggests that risk factors for hypertension amongst PLWH are likely to reflect those of the general population. The substantial prevalence of hypertension with a simultaneous high HIV burden attests to the country's established double burden of infectious and NCDs.

The high prevalence of hypertension noted at enrollment into this study underscores the need for evaluation of NCD comorbidities from the outset to provide comprehensive optimal care. Inadvertently, PLWH with hypertension may develop lifelong sequelae (heart failure, stroke, kidney problems, blindness, etc.) that may impair quality of life when the co-morbidity is not diagnosed early nor adequately managed. Failure to diagnose and treat hypertension early would also affect sustainable economic development because most participants with hypertension were aged 25 – 49 years, which represents the economically active age group in society. Combining care for HIV and hypertension and other NCDs may likely ensure greater compliance with treatment and possibly translate into better clinical outcomes by saving patients from multiple clinic visits for co-morbidities [6, 15].

The association of traditional risk factors such as older age, overweight/obesity, and co-morbidities with the burden of hypertension in PLWH in this study likely emphasizes their role in its development in this vulnerable population which is similar to the general population. This highlights the importance of addressing modifiable risk factors such as overweight/obesity as well as other traditional risk factors (physical inactivity, high salt diets, etc.) that were not examined in this study in PLWH [39, 40]. To address the above risk factors, Nigeria launched a multisectoral action plan to prevent and control NCDs [41]. However, strategies contained in the action plan are in the infancy stage of implementation, thus would require time for proper evaluation of its effects in the health ecosphere.

The prevalence of hypertension in this study was higher compared to studies done among PLWH in Tanzania (5.3% – 11.6%), Uganda (11%), and a global systematic review conducted by Yunan Xu et al. (12.5%) [42–46]. These disparities in hypertension prevalence in PLWH may reflect different levels of traditional risk factors for hypertension in these countries.

Interestingly, a higher CD4 count was significantly associated with prevalent hypertension in this study similar to findings from other studies [44, 47]. The postulated mechanisms focus on HIV viral proteins interacting with the immune system to induce kidney damage, vascular dysfunction, and alterations in sympathetic nervous outflow, which ultimately contribute to hypertension [48]. However, these theories require further elucidation and studies for validity.

Tuberculosis was the most common co-morbidity seen in the cohort and its presence was found to be associated with reduced odds of hypertension. Tuberculosis infection is associated with significant weight loss and is usually accompanied by other opportunistic infections with resultant advanced HIV disease states (WHO Clinical Stage 3 or 4) [49–52]. This significant weight loss and the impact of advanced HIV disease experienced amongst PLWH with co-morbid tuberculosis may substantiate the reduced association with hypertension. Thus, the resultant insufficient immune response could explain the reduced odds of hypertension among ART naïve patients with advanced HIV disease as found in a similar study in Tanzania [53].

Male gender was associated with increased odds of hypertension when compared to females. This finding has been corroborated in other studies [54–56]. Several theories have been postulated to explain the disparity, but the predominant explanations among these are the effects of renin-angiotensin system (RAS), testosterone, and sex hormones that disproportionately increase the odds of hypertension among males [57].

### Strengths and limitations

To our knowledge, this study represents the largest cohort of PLWH for which the prevalence of hypertension has been determined in West Africa. In addition, this study is among the first to determine the age- and sex-standardised prevalence rates for hypertension among PLWH in Nigeria which enables appropriate comparisons with other studies. The different instruments used to measure blood pressure pre- and post-February 2014, i.e., manual sphygmomanometer vs. digital BP monitors could have affected readings. Tobacco use, physical activity, lipid profiles, and dietary consumption were not captured in our analysis as these variables were not collected during clinic consultations. These variables may have provided insight into associated factors for hypertension in the cohort.

While the study sample originated from a single clinic, it is likely representative of the population of PLWH in the Lagos state during the study period. The NIMR HIV clinic has been in operation since 2002, providing care to PLWH from across Lagos state and beyond. Subsidies from the PEPFAR program allowed the care to be free to the end-users, therefore removing financial barriers that could lead to differential enrollment of wealthy people in the clinic.

## Conclusion

Our study showed a substantial burden of hypertension among ART-naïve PLWH at enrollment which was associated with traditional hypertension risk factors of older age, overweight and obesity, and NCD co-morbidities of diabetes and renal disease. This buttresses the need for concomitant care of HIV and hypertension, among other NCD co-morbidities.

## Data Availability

The dataset generated in this study is not publicly available due to the perception of HIV stigma in the country but is available from the corresponding author following due consultations with co-authors on reasonable request.

## Abbreviations

AIC: Akaike Information criterion
AIDS: Acquired Immunodeficiency Syndrome
ART: Antiretroviral therapy
BMI: Body mass index
BP: Blood pressure
CD4: Cluster of differentiation 4
CVD: Cardiovascular disease
DBP: Diastolic blood pressure
HIV: Human Immunodeficiency Virus
LMICs: Low and middle income countries
NAIIS: Nigeria HIV/AIDS Indicator and Impact Survey
NCDs: Non-communicable diseases
NIMR: Nigerian Institute of Medical Research
PEPFAR: US President’s Emergency Plan for AIDS Relief
PLWH: People living with HIV
RAS: Renin angiotensin system
SAMRC: South Africa Medical Research Council
SBP: Systolic blood pressure
SPSS: Statistical Package for Social Sciences
SSA: Sub-Saharan Africa
WHO: World Health Organization
